# Supplementary material for: The transcription factor Foxp1 preserves integrity of an active Foxp3 locus in extrathymic Treg cells
Source: Nat Commun. 2018 Oct 26;9:4473. doi: 10.1038/s41467-018-07018-y (PMC6203760; doi:10.1038/s41467-018-07018-y)
Supplement: Supplementary file 1 — Supplementary information [file 41467_2018_7018_MOESM1_ESM.pdf]

Supplementary information

The transcription factor Foxp1 preserves integrity of an active *Foxp3* locus in extrathymic Treg cells

Ghosh et al.

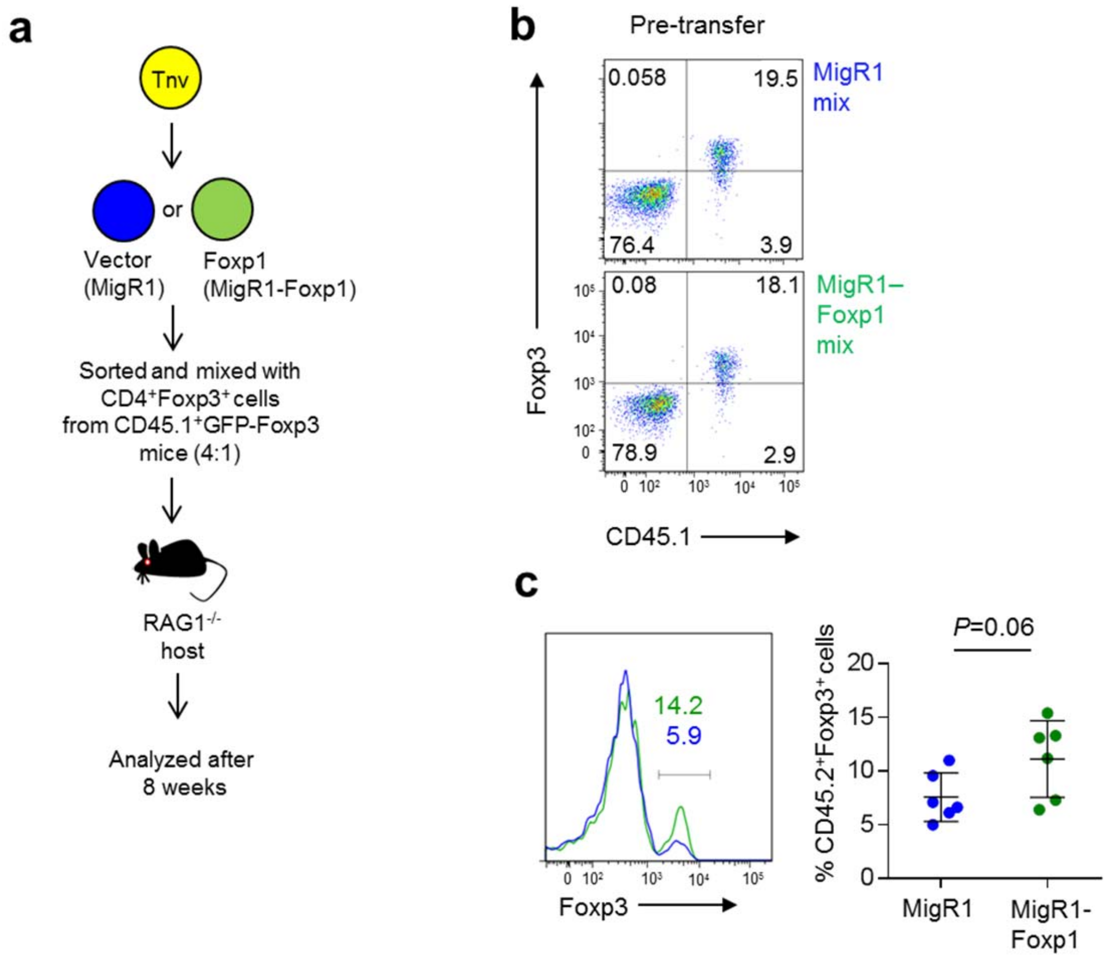

Supplementary figure 1. Overexpression of Foxp1 in CD4^{+} T-cells results in enhanced yield of iTreg *in vivo.*

(a) Experimental scheme. Naïve T-cells sorted from *Foxp3*$^{\text{IRES-Thy1.1}}$ mice were transduced with cDNA encoding full length Foxp1 or empty vector. Transduced cells were sorted based on the reporter green florescent protein (GFP) expression and adoptively transferred in *RAG1*$^{-/-}$ hosts along with allelically marked Treg cells from *Foxp3*^{GFP} mice. *In vivo* conversion to iTreg cells within the transferred naive populations were analyzed after 8 weeks. **(b)** Flow cytometric analysis of the cell mixture before transfer. **(c)** Representative FACS plot and frequencies of *in vivo* generated iTreg cells determined by CD4^{+}Foxp3^{+} cells within CD45.2^{+} compartment isolated from Large intestine Lamina propria (LI-LP) of indicated recipient mice. Data are representative of two independent experiments. Each symbol represents a single recipient mouse, and the data are shown as ±s.d.

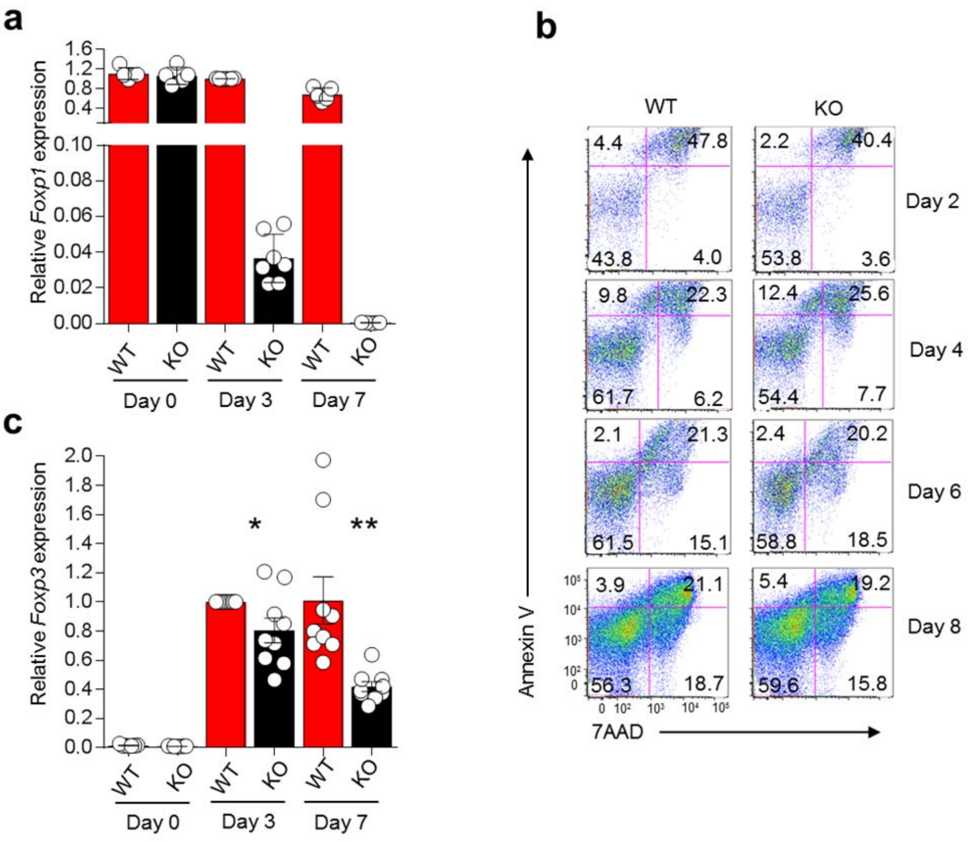

Supplementary figure 2. ***In vitro*** **iTreg differentiation of Tnv cells derived from** ***Foxp1***$^{\mathbf{f/f}}$***Foxp3***$^{\mathbf{IRES\text{-}YFP\text{-}Cre}}$ **is not associated with enhanced cell death.**

(a) Real time qPCR analysis of *Foxp1* mRNA in WT and KO *in vitro* induced iTreg cells at indicated time points after TGFβ treatment. **(b)** Comparison of cell death between WT and KO iTreg cells determined by Annexin-V and 7AAD staining, at indicated time points during the course of the experiment. **(c)** Real time qPCR analysis of *Foxp3* mRNA during the course of the experiment. At all points live cells were sorted and total RNA was prepared for further processing. Relative enrichment over *Hprt1* is shown. Data represents two to four independent experiments. *P < 0.05, **P < 0.01, (Student’s t-test, error bars, s.e.m).

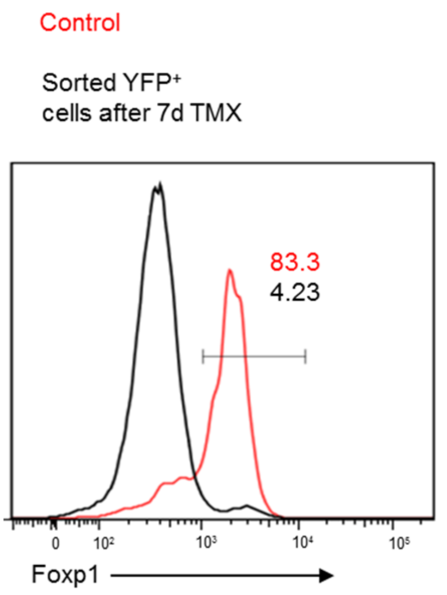

Supplementary figure 3. Tamoxifen induced YFP$^+$ cells efficiently delete Foxp1 expression *in vivo*

Intracellular staining of Foxp1 in YFP$^+$ Treg cells sorted 7 days after Tamoxifen treatment to *Foxp1*$^{f/f}$*Foxp3*$^{eGFP\text{-}Cre\text{-}ERT2}$*R26Y* mice. YFP$^+$ cells sorted from *Foxp1*$^{+/+}$*Foxp3*$^{eGFP\text{-}Cre\text{-}ERT2}$*R26Y* was used as control.

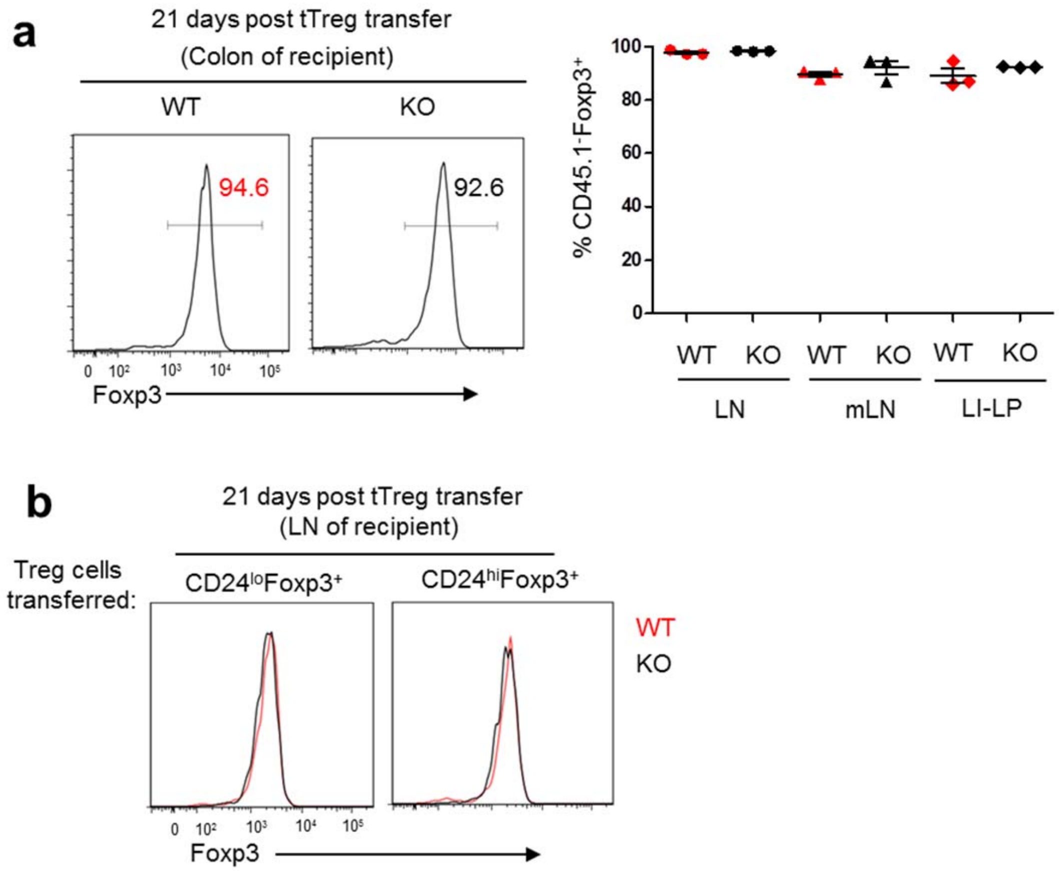

Supplementary figure 4. Foxp1 deficient tTreg cells maintain Foxp3 expression.

(a) Histogram and quantification showing stability of Foxp3 expression in tTreg cells sorted from thymus of WT and KO animals 21days after co-transfer to *RAG1*$^{-/-}$ recipients. LN; lymph nodes, mLN; mesenteric lymph nodes, LI-LP; large intestinal lamina propria. **(b)** Similar experiment as explained in **(a)** performed to compare stability between CD24^{lo} (mature) and CD24^{hi} (immature) tTreg cells. Data is representative of two independent experiments.

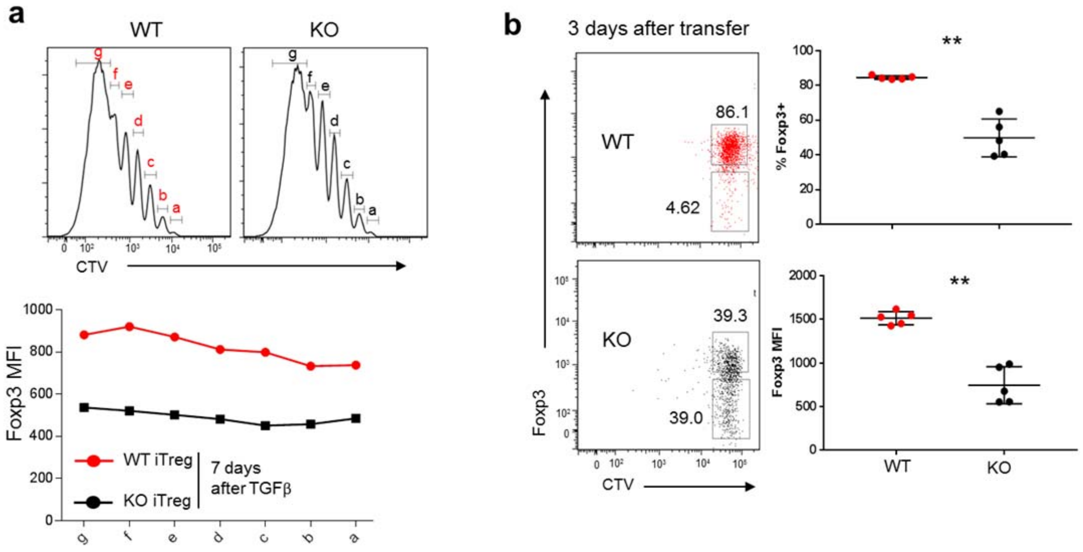

Supplementary figure 5. Foxp1 maintains optimum expression of Foxp3 in a cell cycle independent manner.

(a) Sorted Tnv-cells from WT and KO mice were labeled with Cell Trace Violet (CTV), activated and induced with TGFβ *in vitro*. 7 days after induction, numbers of cell divisions ("a" through "g") were visualized based on CTV dilution and Foxp3 MFI was measured and plotted corresponding to each cell division. One of three independent experiments is shown. **(b)** Sorted Tnv-cells from WT and KO mice were differentiated to iTreg cells *in vitro* for 3 days when they were sorted, labeled with CTV and co-transferred in *RAG1*$^{-/-}$ hosts along with allelically marked CD4^{+} T-cells derived from *CD45.1*$^{+}$*Foxp3*^{GFPKO} mice. 3 days post-transfer, mice were sacrificed and Foxp3 expression was analyzed. Representative FACS plots within CD4^{+}TCRβ^{+}CD45.2^{+} gate and quantification of percentage cells retaining Foxp3 and MFI of Foxp3 between undivided WT and KO populations are shown in the left and right respectively. Data is representative of two independent experiments. *$P < 0.05$, **$P < 0.01$, (Student's t-test, error bars, s.d.).

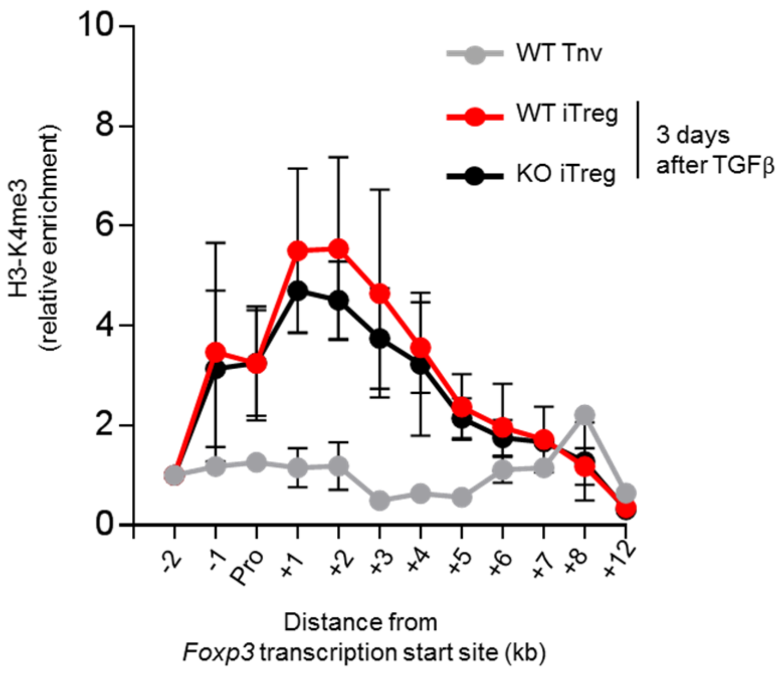

Supplementary figure 6. Comparable H3-K4me3 modification of *Foxp3* locus in WT and KO mice after three days of TGFβ treatment.

Relative enrichment of H3-K4me3 throughout the *Foxp3* locus in sorted Tnv-cells from WT mice before induction and WT- or KO-derived iTreg cells at day 3 from *in vitro* culture after TGFβ treatment. Relative distances (kb) from Foxp3 transcription start site (TSS) of primer probes are indicated in the X-axis. Pro: Promoter. Data represents one of three independent experiments.

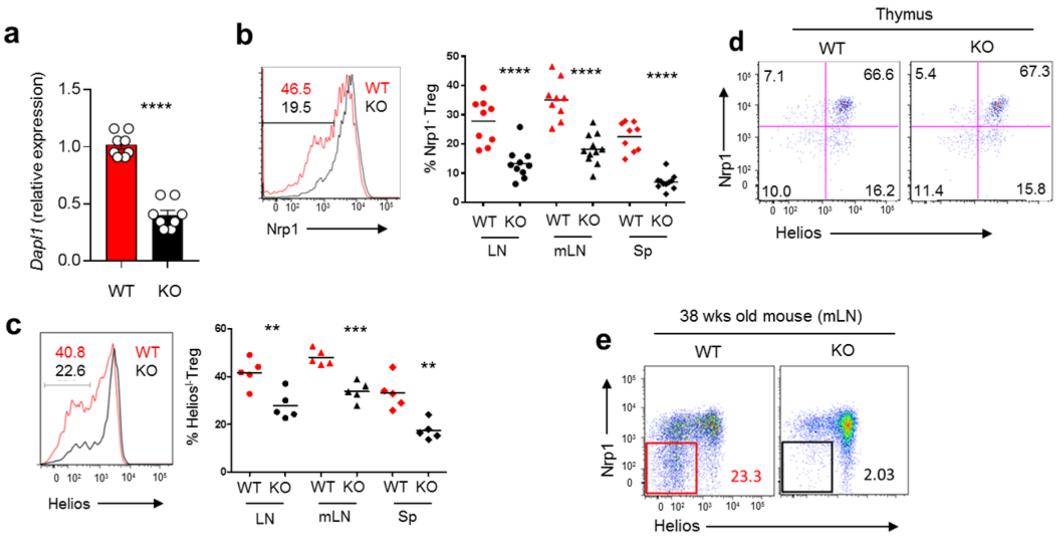

Supplementary figure 7. Reduced iTreg compartment in the secondary lymphoid organs of *Foxp1*$^{f/f}$*Foxp3*$^{IRES\text{-}YFP\text{-}Cre}$ mice.

(a) Real-time PCR analysis of *Dapl1* mRNA normalized against *Hprt1* mRNA in sorted CD4$^+$Foxp3$^+$ Treg cells from WT and KO mice. **(b)** Representative FACS plot and quantification showing Nrp1$^-$ iTreg compartment in WT and KO mice. **(c)** Representative FACS plot and quantification showing Helios$^-$ iTreg compartment in WT and KO mice. **(d)** Representative FACS plot showing comparable Nrp1$^-$Helios$^-$ population within CD4SDFoxp3$^+$ gate in the thymus of WT and KO mice. **(e)** Representative FACS plot showing dramatically compromised status of Nrp1$^-$Helios$^-$ iTreg population in the mLN of aged (38 weeks old) KO mice compared to WT. Data is representative of at 3-5 independent experiments. **P < 0.01, ***P < 0.0001, ****P < 0.0001, (Student's t-test, error bars, s.e.m).

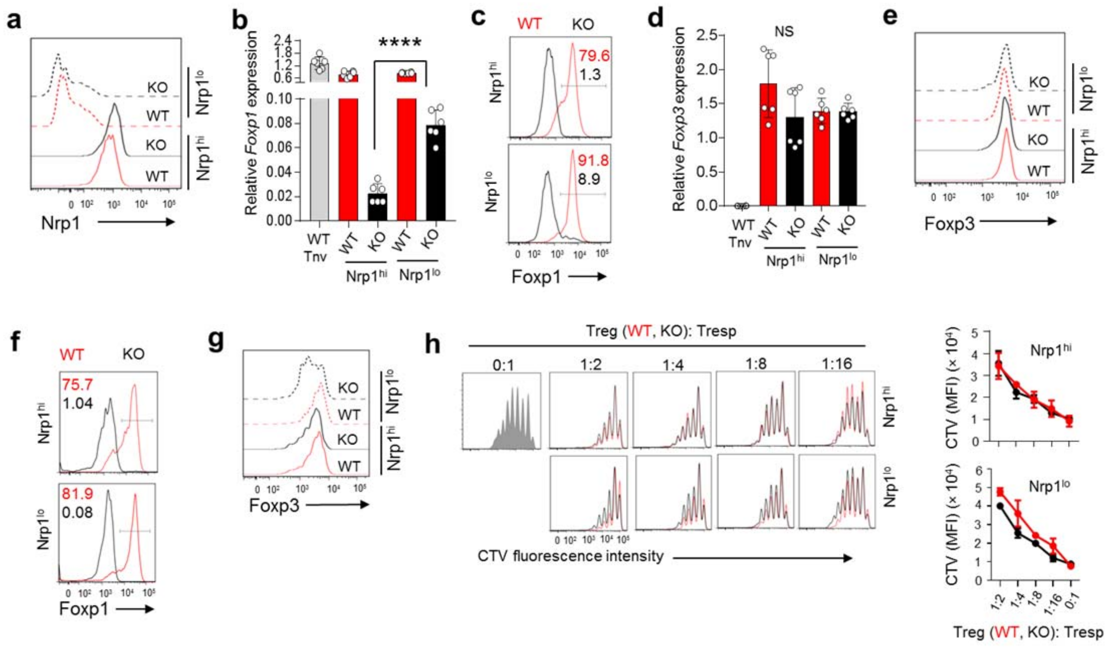

Supplementary figure 8. Residual $Foxp3^{+}Nrp1^{lo}$ cells $\textit{Foxp1}^{f/f}\textit{Foxp3}^{IRES\text{-}YFP\text{-}Cre}$ mice represent 'recently generated' iTreg cells.

(a) Post-sort Nrp1 staining on $Nrp1^{lo}YFP^{+}$ and $Nrp1^{hi}YFP^{+}$ cells from WT or KO mice. **(b)** Real time qPCR analysis of Foxp1 in each cell types as mentioned in (a). ****$P < 0.0001$, (Student's t-test, error bars, s.e.m). **(c)** Intracellular staining to determine Foxp1 protein expression in the indicated $Nrp1^{hi}$ and $Nrp1^{lo}$ cell populations. **(d-e)** Real-time qPCR analysis (d) and intracellular FACS staining (e) to determine Foxp3 mRNA and protein expression within the indicated sorted cell populations. **(f-g)** Sorted $Nrp1^{hi}$ and $Nrp1^{lo}$ Treg cells from WT and KO mice were mixed with allelically marked $CD45.1^{+}$ Tnv cells and cultured in the presence of plate-bound anti-CD3/CD28 antibodies and 100 IU/ml IL2 for 3 days. Expression of Foxp1 (f) and Foxp3 (g) within the $CD45.1^{-}$ gate were determined by FACS staining. **(h)** *In vitro* assay, illustrating dose-dependent suppression of T-responder (Tresp) cell proliferation by the indicated populations of Treg cells. Histogram represents CTV dilution of labelled sorted Tnv cells alone or co-cultured with indicated sorted Treg cell populations at mentioned Treg: Tresponder (Tresp) ratios (left). Cumulative data expressed as CTV MFI of responder $CD4^{+}$ cells is shown in the right panel. One of two independent experiments, which gave similar results, is shown.

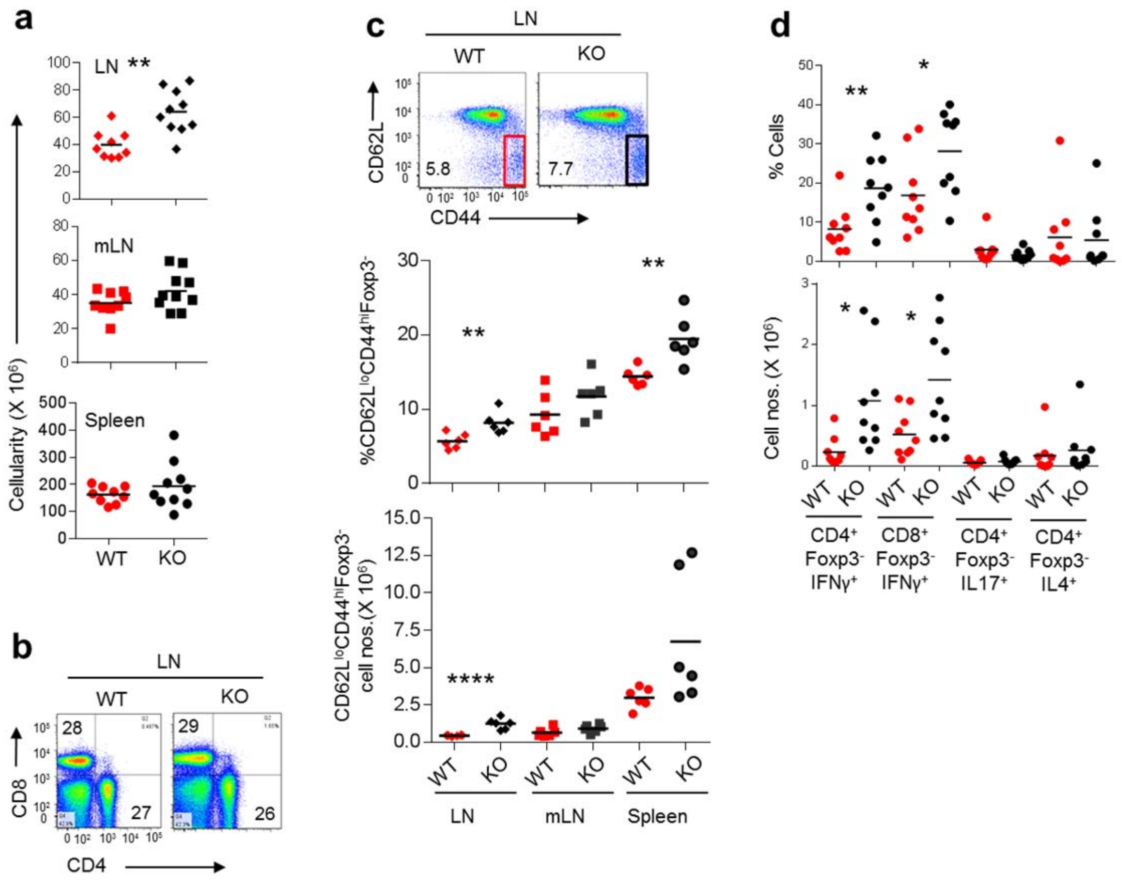

Supplementary figure 9. Moderate inflammation within secondary lymphoid organs in *Foxp1*$^{f/f}$*Foxp3*$^{IRES\text{-}YFP\text{-}Cre}$ mice.

(a) Total cellularity within indicated secondary lymphoid organs in 6-8 weeks old WT and KO mice. **(b)** Representative FACS plots showing $CD4^+$ and $CD8^+$ T-cell compartments within lymph nodes of WT and KO mice. **(c)** Representative FACS plots and quantification of effector memory T-cells as represented by $CD44^{hi}CD62L^{lo}$ populations within $CD4^+Foxp3^-$ gate, in WT and KO mice. **(d)** Quantification of intracellular cytokine staining within indicated populations derived from lymph nodes of WT and KO mice. Data are representative of at least three independent experiments. **$P < 0.01$, **$P < 0.01$ (Student's t-test).

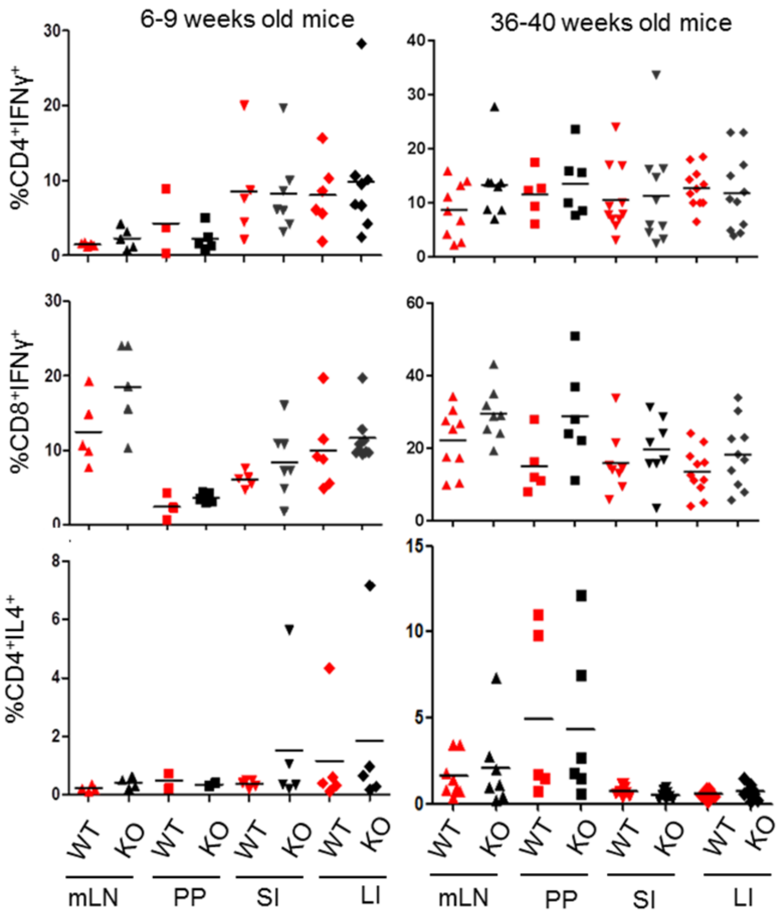

Supplementary figure 10. Cytokine profile in GALT of young and aged *Foxp1*$^{f/f}$*Foxp3*$^{IRES\text{-}YFP\text{-}Cre}$ mice compared to littermate controls.

Intracellular cytokine staining in cells isolated from GALT tissues in young and aged WT and KO mice. Data is representative of at least three independent experiments. mLN; mesenteric lymph node, PP; Peyer's patches, SI; small intestine, LI; large intestine.

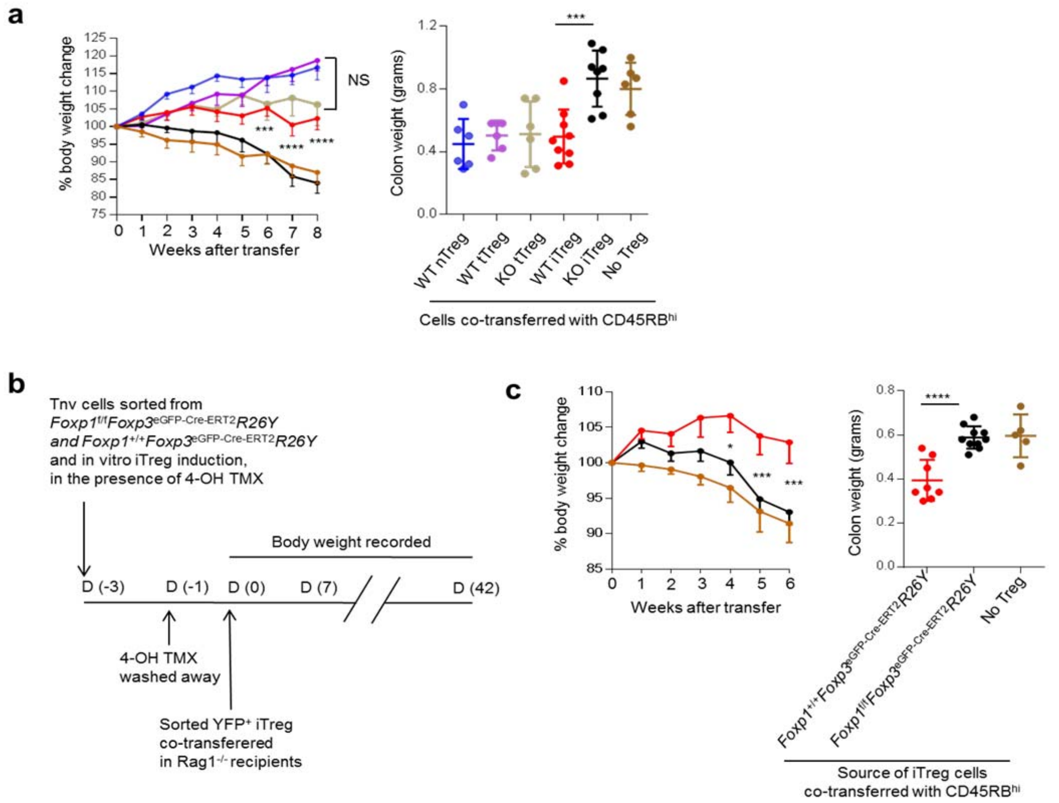

Supplementary figure 11. *Foxp1*$^{f/f}$*Foxp3*$^{IRES\text{-}YFP\text{-}Cre}$ tTreg cells but not iTreg cells can protect hosts from CD45RB^{hi} T-cell transfer induced colitis.

(a) Percentage body weight over time and colon weight of representative *RAG1*$^{-/-}$ mice viable at the end of the experiment, which received either sorted pure CD45.1^{+}CD45RB^{hi} naïve T-cells (disease control) or combined with total wild type, Foxp1-sufficient (WT) or Foxp1^{-} deficient (KO) thymic or *in vitro* induced Treg cells. Data is representative of three independent experiments (n= 6 for CD45RB^{hi} only, co-transfer of total Treg and thymic treg groups and n=13 for induced Treg co-transferred groups). Mean ± s.e.m.***P < 0.001, ****P < 0.0001 between CD45RB^{hi} + WT iTreg and CD45RB^{hi} + KO iTreg. NS= non-significant (two way ANOVA, Bonferroni post-test). **(b)** Experimental plan. Double FACS sorted >99% pure Tnv cells from *Foxp1*$^{f/f}$*Foxp3*$^{eGFP\text{-}Cre\text{-}ERT2}$*R26Y* or control *Foxp1*$^{+/+}$*Foxp3*$^{eGFP\text{-}Cre\text{-}ERT2}$*R26Y* mice were activated *in vitro* for 3days with 1ug/ml of plate bound anti-CD3/CD28 simultaneously in the presence of 0.3µM 4-OH Tamoxifen and TGFβ with 50 IU/ml of IL2. After 2 days, cells were washed off 4-OH TMX. 3days post induction YFP^{+} cells were sorted and co-transferred along with CD45RB^{hi} cells in RAG1$^{-/-}$ recipients. The disease control group contained animal having only CD45RB^{hi} cells injected in them. Body weight was measured until 6th week after cell transfer **(c)** Percentage body weight loss over time (left panel) and colon weight (right) of indicated groups. Two way ANOVA with Bonferroni post-test was applied and statistically significant differences in mean values is considered at *P < 0.05,***P < 0.001. Data is representative of two experiments n= 5 for CD45RB^{hi} only (no Treg), n=8 and 10 for induced Treg co-transferred groups derived from *Foxp1*$^{+/+}$*Foxp3*$^{eGFP\text{-}Cre\text{-}ERT2}$*R26Y* and *Foxp1*$^{f/f}$*Foxp3*$^{eGFP\text{-}Cre\text{-}ERT2}$*R26Y* mice respectively.

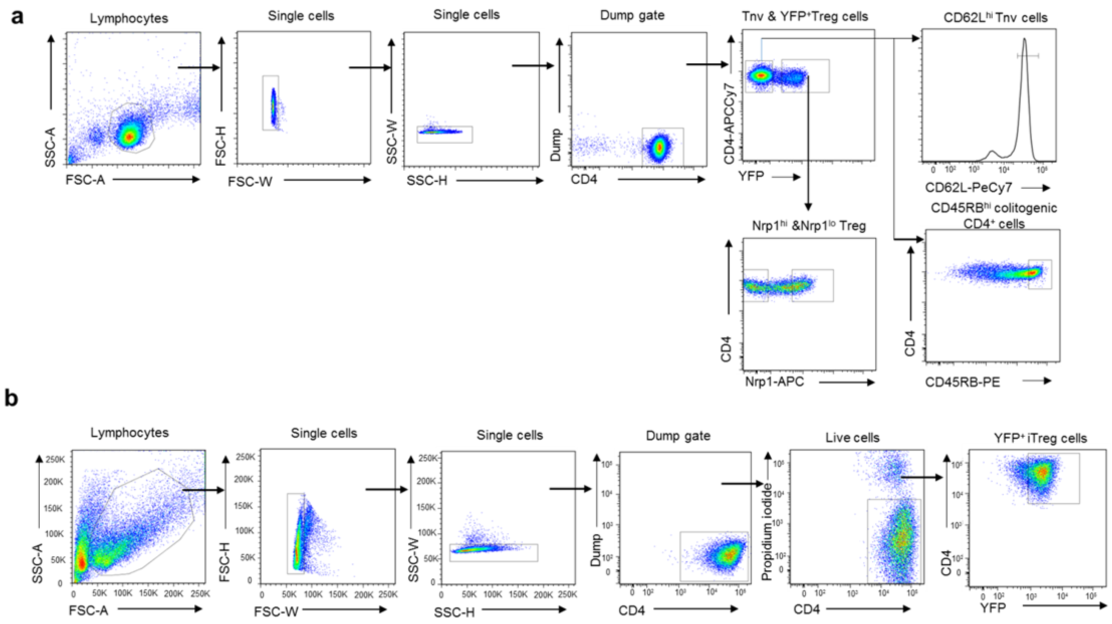

Supplementary figure 12. Gating strategy for sorting

Gating strategy used in all experiments for sorting of **(a)** CD4^{+}CD62L^{hi}YFP^{-} Tnv-cells, CD45RBhi colitogenic CD4^{+} T cells, YFP^{+}Nrp1^{hi} and YFP^{+}Nrp1^{lo} Treg cells, **(b)** YFP^{+} Treg cells induced for 3 days in *in vitro* culture.

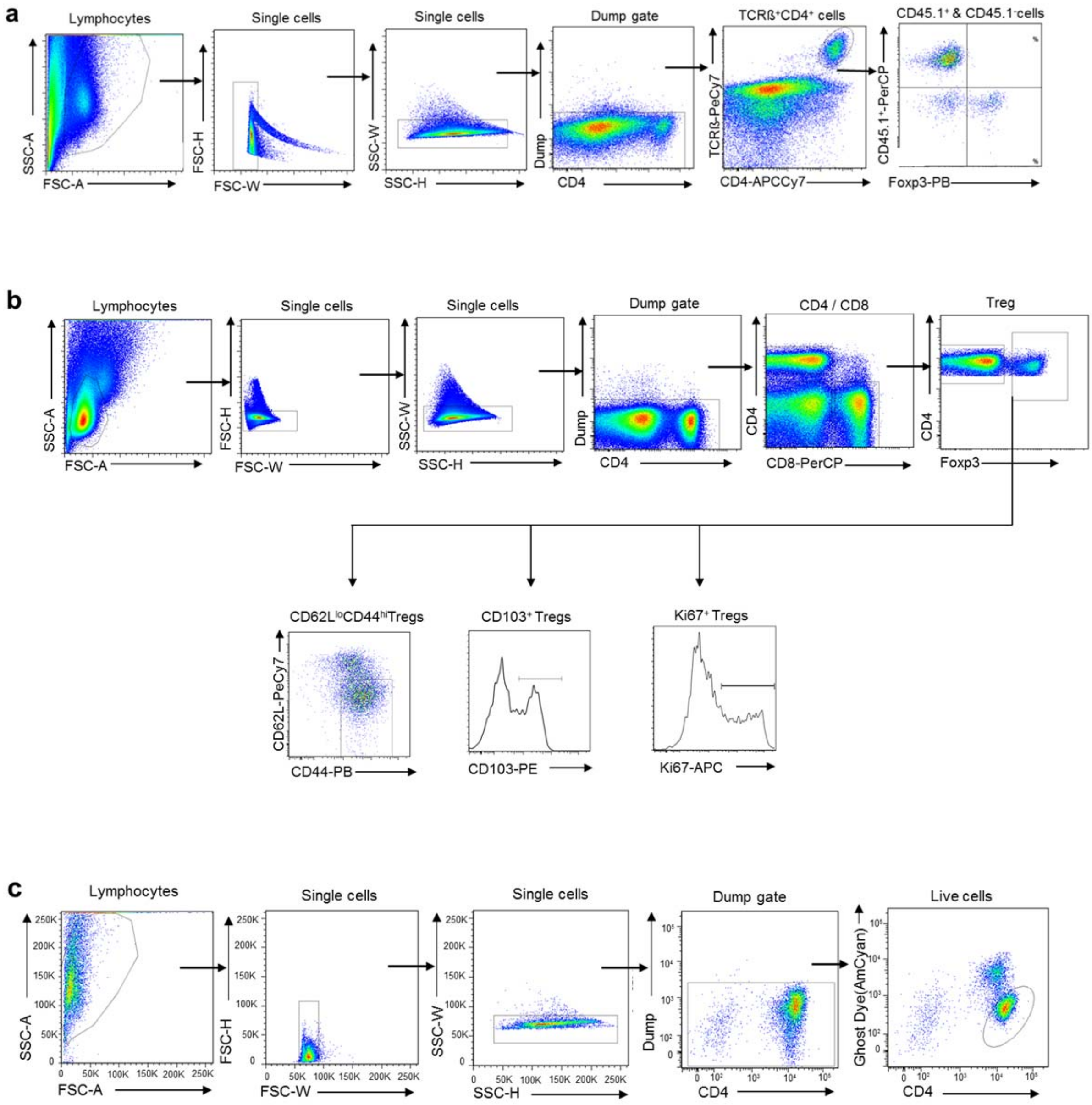

Supplementary figure 13. Gating strategy for FACS analyses

Gating strategy used for analysis of **(a)** all the experiments where allelically marked $CD45.1^{+}$ Tnv-cells were co-transferred with Treg cells, **(b)** Treg activation and proliferation markers as shown in Fig. 6b and 6d) and **(c)** live cells in all the *in vitro* cell culture experiments.

Supplementary Table 1: Sequence of primers used in this study

Bisulfite sequencing

DRO834	TGTTAGGGTATTAAAGGTTGGAAGTT	Forward	-1.5
DRO835	CCAATTTTCCTAAAACCAACAATAT	Reverse	
DRO822	ATTTGAATTGGATATGGTTTGT	Forward	CNS2
DRO823	AACCTTAAACCCCTCTAACATC	Reverse	
DRO836	TATATTTTTAGATGATTTGTAAAGGGTAAA	Forward	Promoter
DRO837	ATCAACCTAACTTATAAAAAACTACCACAT	Reverse	

RNA analysis

DRO747	GCAGTGAAAGCTGGAGGGATGCG	Forward	DapL1
DRO748	TGTGCCGTGTGAACTGTCGCTG	Reverse	
DRO790	CGATAGAAGCACAGCTCAAT	Forward	Foxp1
DRO791	ACATGCAGGTGGGTCATCAT	Reverse	
DRO793	TTCCTTCCCAGAGTTCTTCC	Forward	Foxp3
DRO794	CTCAAATTCATCTACGGTCCA	Reverse	
DRO282	AGCCTAAGATGAGCGCAAGT	Forward	HPRT
DRO283	TTACTAGGCAGATGGCCACA	Reverse	

ChIP

DRO862	GAGCCGGTCTGTGCCAAAT	Forward	upstream 2 kb
DRO863	GACTCCTCTGGAACTTGATGTTTGT	Reverse	
DRO860	CTGAGGTTTGGAGCAGAAGGA	Forward	upstream 1kb
DRO861	TCTGAAGCCTGCCATGTGAA	Reverse	
DRO803	CCTCCAACGTCTCACAAACA	Forward	Promoter
DRO804	TCAATGAGATAACAGGGCTCA	Reverse	
DRO846	TTCCTCCCGCTCTCTGACTCT	Forward	downstream 1kb
DRO847	AAGCGCCAGTTGTGTACAAATATC	Reverse	
DRO848	ACTTAGTTTATGAGCATGCATGTTCTTC	Forward	downstream 2kb
DRO849	TGAGATCCCACACCATCTTCTG	Reverse	
DRO850	TGTCCTGCACTGTTCCTCATG	Forward	downstream 3kb
DRO851	AGAGTAGAAAACCGTGGCAGAGA	Reverse	
DRO852	GACCCAGGAGGCCATTAACA	Forward	downstream 4kb
DRO853	AGATTTGGCCCCATGCTATG	Reverse	
DRO587	GTTGCCGATGAAGCCCAAT	Forward	downstream 5kb/CNS2
DRO588	ATCTGGGCCCTGTTGTCACA	Reverse	
DRO854	AGCCCCAGACATGATAGCAAA	Forward	downstream 6kb
DRO855	TTGGGCATGTAGCTTCTGAGAA	Reverse	
DRO856	GTCATTGGAATAAAAAGATGAGAAGAGA	Forward	downstream 7kb
DRO857	CCAGTACCCCCTGCACTCTGT	Reverse	
DRO858	AATGAATGAGACACAGAACTATTAAGATGA	Forward	downstream 8kb
DRO859	CAGACGGTGCCACCATGAC	Reverse	
DRO840	GGCTACAATGAAATGACAAGCTTAAG	Forward	downstream 12kb
DRO841	TGGCTACGATGCAGCAAGAG	Reverse	
DRO805	TCTCCAGGCTTCAGAGATTCAAGG	Forward	CNS3
DRO806	ACAGTGGGATGAGGATACATGGCT	Reverse	
DRO619	CAGCTGGAACAGCCTTGGAA	Forward	Gmpr
DRO620	AAATGTCAAGGCCCCTGTGA	Reverse	
DRO795	AGCCTTTCATGGGCTATCACTCCA	Forward	IL7r enhancer
DRO796	GAGCAAACTAGCACATGCTGTACC	Reverse	
